# Laparoscopic ureteroneocystostomy with bladder flap for benign ureteral stenosis: our initial experience

**DOI:** 10.1038/s41598-024-52497-3

**Published:** 2024-01-23

**Authors:** Zhaolin Zhang, Ruohui Huang, Tianpeng Xie, Qingming Zeng, Linwei Liu, Xiaofeng Zou, Guoxi Zhang, Yuanhu Yuan, Gengqing Wu, Zhihua He, Yuting Wu, Hui Xu

**Affiliations:** Department of Urology, First Affiliated Hospital of Gannan Medical University, No. 128, Jinling Road, Ganzhou, 341000 Jiangxi China

**Keywords:** Urology, Ureter

## Abstract

To present our experience with laparoscopic ureteroneocystostomy with bladder flap (LUCBF) for treating benign ureteral stenosis and evaluate its feasibility and efficacy. The clinical data of 27 patients with benign ureteral stenosis who underwent LUCBF were retrospectively analyzed. After identification and excision of the ureteral stenosis segment, the healthy ureteral stump was dissected and incised longitudinally. A U-shaped or spiral bladder flap was harvested from the anterolateral bladder wall for ureteroplasty. All patients underwent LUCBF successfully, including 14 patients were combined with psoas hitch technique, between 90 and 220 min (median, 155 min). The median length of ureteral defect was 6 cm (range, 5–17 cm). The median blood loss was 40 ml (20–150 ml). The median indwelling time of double-J stent was 8 weeks (range, 4–8 weeks). Five patients (10.6%) suffered postoperative complications during the follow-up period (range, 12–48 months), including fever, hematuria, urinary tract infection and recurrent stenosis. The success rate was 96.3% (26/27). Patients with long ureter defects had longer operative time and more blood loss than short ureter defects. LUCBF was a safe and feasible technique for benign ureteral stenosis. Long ureter defect was related to longer operative time and more blood loss.

## Introduction

Iatrogenic injury is the most common causative factor of ureteral stenosis, and other factors include ureteral calculi, radiological damage, and ureteral mass^1^. The distal ureter is most susceptible to damage, followed by the middle and proximal segments^2^. The choice of therapeutic strategy for ureteral stenosis is based on the stenosis site, length of the ureter lesion, patient status, and surgeon preference.

Endoscopic procedures are more commonly used for short ureteral strictures; however, for long and complex stenosis, ureteral reconstruction surgery is strongly recommended^3^. For distal ureter stenosis, effective definitive reconstructive methods, including ureteroneocystostomy, psoas hitch (PH) technique and Boari bladder flap, are performed^4^. For the stenosis located at the middle or proximal ureter, transureteroureterostomy or pyeloplasty is applied for short stenosis with a good success rate. However, this approach is inappropriate for patients with ureteral avulsion or long stenosis at middle or proximal ureter. Boari flap constriction, bowel interposition or renal autotransplantation are suggested^5^.

In our study, we presented our experience with laparoscopic ureteroneocystostomy with bladder flap (LUCBF) for ureteral stenosis caused by benign ureteral lesions, and summarized the long-term outcomes.

## Materials and methods

### Data collection

The medical records of patients with unilateral ureteral stenosis caused by benign ureteral lesions who underwent LUCBF at the First Affiliated Hospital of Gannan Medical University between July 2019 and July 2022 were retrospectively reviewed. The exclusion criteria were as follows: (a) combined with urinary carcinoma; (b) open reconstructive surgery; (c) previous urinary diversion surgery, including cutaneous ureterostomy, ileal conduit, or neobladder after radical cystectomy. Finally, 27 patients were included, including 14 patients underwent LUCBF combined with PH technique. The surgeries were performed by a single senior surgeon with rich experience in urologic reconstruction and expertise in laparoscopic manipulations.

Preoperatively, various radiological examinations encompassing urinary ultrasound, intravenous urography (IVU), retrograde pyelogram (RGP), computed tomography (CT) urography or magnetic resonance urography (MRU) were used to assess the degree of hydronephrosis, the location and the length of ureteral stenosis. Routine urinalysis and urine culture were performed and appropriate antibiotics were administered preoperatively. Routine blood and serum tests were performed. Preoperative demographic characteristics including gender, age, body mass index, preoperative symptoms, operative side, location of the ureteral lesion, etiology of ureteral stenosis, and hydronephrosis were obtained from the medical records.

### Ethics approval

Ethical approval for the study protocol was obtained from the Ethical Committee of the First Affiliated Hospital of Gannan Medical University (proof number: 2023032702), and the study was performed in accordance with the Declaration of Helsinki (as revised in 2013).

### Informed consent

Written informed consent was obtained from all participants included in the study.

### Surgical techniques

All patients were explicated with all alternative therapeutic strategies, and informed consent were obtained before the operation. After general anesthesia, the patient was placed in the supine position with the affected side was elevated at 30°-45°. A 20 Fr three-way Foley urethral catheter was inserted before the pneumoperitoneum was established. A 10 mm trocar was placed near the umbilicus for camera access, a 5 mm trocar was placed below the umbilicus along the midclavicular line on the contralateral affected side, and a 10 mm trocar was placed in the lower abdomen. For upper ureter stenosis, another 10 mm trocar was located on the affected side.

The colon was freed along the Toldt line to expose the retroperitoneal space. The ureter was identified at the crossing point with the ipsilateral common iliac artery. For ureter lesion was located above the iliac artery, the ipsilateral renal pelvis and renal lower pole were first separated as anatomical markers to identify the proximal ureter. The ureter was then freed anterogradely until reaching the ureteral stenosis segment. Careful ureterolysis was needed to avoid the devascularization of ureter and to protect healthy ureteral tissue. The ureter proximal to the stenosis segment was transected and dissected with scissors until the healthy pink ureteral mucosa was exposed. The posterior wall of the ureter was incised longitudinally to 2.0 cm to prepare for anastomosis.

The bladder was visually distended after it was filled with 300 mL of normal saline (0.9%). The anterior peritoneum overlying the bladder and the anterior and ipsilateral walls of the bladder were adequately mobilized. The length of the ureteral defect was measured intraoperatively from the distal end of the healthy ureter to the bladder. A U-shaped or spiral bladder flap with a width of 4–5 cm at the base and 2–3 cm at the tip was designed on the anterolateral bladder wall. The length of the flap was designed to be 2.0 cm longer than the ureteral defect. The length/width ratio was 3–4:1. The base of the flap was located at the dome and the tip was near the bladder neck. For long ureteral defect, especially for proximal or middle ureteral stenosis, the spiral bladder flap was harvested. The flap was harvested from the anterior wall of the contralateral bladder to the lateral wall, and then to the parietal wall, ending at the ipsilateral posterior wall. The base of the flap extended up to 6–8 cm and the PH technique was used, the flap was sutured to the psoas fascia. The entire thickness of the detrusor muscle of the posterior bladder flap was fixed to the psoas muscle.

**Figure 1:**
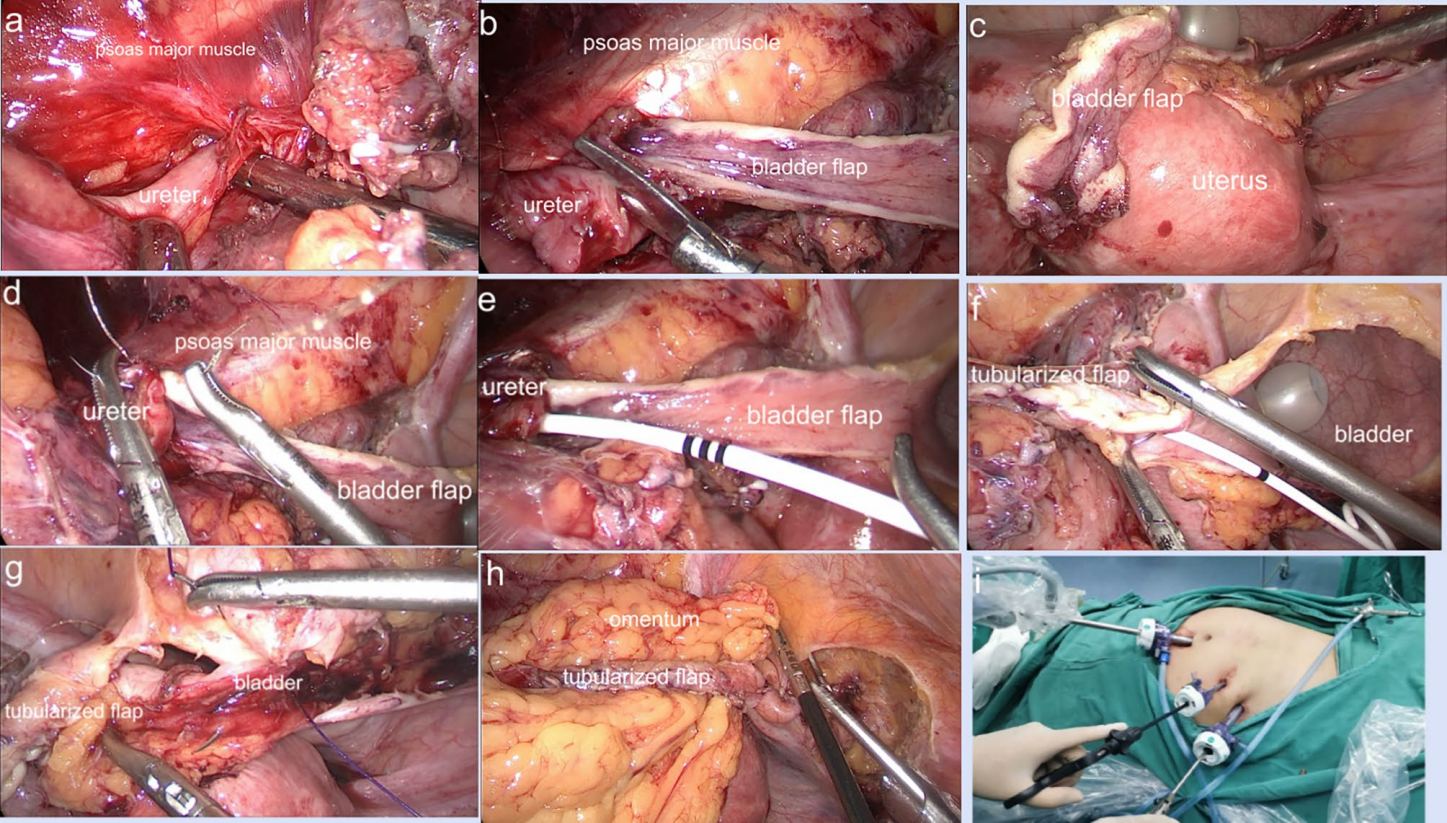
Surgical procedures. (**a**) separated the ureter; (**b**) posterior wall of ureter was incised longitudinally; (**c**) bladder flap was harvested; (**d**) anastomosis of ureter posterior wall and apex of flap; (**e**) a 7 Fr/26 cm double J stent was inserted; (**f**) tubularized flap; (**g**) bladder incision was closed; (**h**) pedicled omentum wrapped around anastomosis; (**i**) trocars placement.

Tension-free anastomosis of the ureter posterior wall and apex of the flap was performed using a full-thickness style with 4–0 absorbable sutures in an interrupted fashion, and the suture needles were 1/2 circular, tapered and 17 mm long. After a 7 Fr/26 cm double J stent was inserted across the ureter-flap anastomosis site, a complete circumferential anastomosis was performed between the lateral edge of the flap and the ureteral margin using discontinuous sutures. The residual flap was tubularized with continuous 4–0 absorbable sutures. The bladder incision was closed with continuous 2–0 or 3–0 absorbable sutures, and both the suture needles were 1/2 circular, tapered and 26 mm long. Normal saline was reinjected into the bladder to check for anastomotic leakage. In patients with ureterovaginal fistulas, fistula dissection and vaginal stratified suturing were performed. Adjacent well-vascularized pedicled omentum was harvested to wrap around the ureterovesical anastomosis and tubularized flap in a tension-free condition, also for vaginal anastomosis if present. A 20 Fr drainage tube was inserted and the previous urethral catheter continued to indwell in bladder. The details of the surgical procedure are shown in Fig. 1a–i.

### Postoperative management and follow-up

The drainage tube was removed when the total volume was ≤ 20 ml/d. The urethral catheter was removed at 14 days after surgery. The double J stent was removed at 4–8 weeks after surgery. During the follow-up period, patients were recommended to be reassessed at 3, 6, and 12 months postoperatively and then annually. The protocols included clinical examination, laboratory examination and appropriate imaging studies including ultrasonography at each return visit, IVU or CT or MRU were performed at 3, 6, 12 months after surgery and yearly thereafter. Surgical success was defined as improvement or stabilization of hydronephrosis, relief of symptoms, and no recurrence of ureter stenosis.

### Statistical analysis

Statistical analysis was executed using SPSS 21.0 Statistics (IBM, Armonk, NY). The median and range were used for the quantitative variables, and the Mann–Whitney U test was used to compare groups. Qualitative variables were expressed as percentages (%) or numbers(n), and differences between groups were analyzed using the Chi-squared test or Fisher's exact test. A two-tailed P value less than 0.05 was considered as an indicator of statistical significance.

## Results

The demographic characteristics and preoperative data are summarized in Table 1. A total of 27 patients—10 male patients and 17 female patients —had a median age of 46 years (range, 21–81 years). Of these patients, 16 patients had a left-sided ureter defect and 11 patients had a defect on the right side. Flank and/or abdominal pain were the most common symptoms, followed by no clinical symptoms, vaginal leakage, fever, or hematuria. Nine patients suffered stenosis secondary to ureteral holmium laser lithotripsy, and six patients were diagnosed with ureterovaginal fistula after hysterectomy. There were two patients with previous ureterovesical reimplantation and one patient with previous ureteroureterostomy who experienced recurrence of ureteral stenosis. Ureteral injury occurred during ureteroscopy in three patients, two of whom had ureteral avulsion. Non-iatrogenic causes included ureteral polyps in three patients and impacted calculi in one patient. Preoperatively, six patients underwent preoperative ureteral stent placement, and two patients underwent percutaneous nephrostomy.

**Table 1:** Clinical outcomes of patients.

Variables	Total
Total number, (n)	27
Age (years), median (range)	46 (21–81)
Gender, n (%)	
Male	10 (37.0%)
Female	17 (63.0%)
Body mass index (kg/m^2^), median (range)	23.43 (17.76–27.55)
Operative side, n (%)	
Left	16 (59.3%)
Right	11 (40.7%)
Preoperative symptoms, n (%)	
Flank or/and abdominal pain	15 (55.6%)
Fever	3 (11.1%)
Vaginal leak	4 (14.8%)
Hematuria	1 (3.7%)
No symptoms	7 (26.0%)
Aetiology of ureteral stenosis	
Urological surgery	
Ureteral Holmium laser lithotripsy	9 (33.3%)
Ureterovesical reimplantation	2 (7.4%)
Ureteroureterostomy	1 (3.7%)
Ureteroscopy	3 (11.1%)
Gynecological surgery	
Laparoscopic hysterectomy	6 (22.2%)
Cesarean section	2 (7.4%)
Ureteral polyp	3 (11.1%)
Impacted calculi	1 (3.7%)
Ureteral lesion location, n (%)	
Proximal ureter	6 (22.2%)
Middle ureter	6 (22.2%)
Distal ureter	15 (55.6%)
Hydronephrosis	
Mild	11 (40.7%)
Moderate	7 (26.0%)
Gross	9 (33.3%)
Preoperative management	
None	19 (70.4%)
Ureteral stent placement	6 (22.2%)
Percutaneous nephrostomy	2 (7.4%)
Preoperative serum creatinine (μmol/L), median (range)	88.00 (46–393)
Preoperative eGFR (ml/min*1.73 m^2^), median (range)	77.86 (11.57–134.17)

*eGFR* estimated glomerular filtration rate.

All surgical procedures were successfully completed without intraoperative complications, and none were converted to open surgery or other reconstructive treatments. All patients underwent LUCBF, including 14 patients who underwent surgery in combination with the PH technique during surgery. The median length of the ureter defects was 6 cm (range, 5–17 cm) and the duration of surgery ranged from 90 to 220 min (median, 155 min). The median blood loss was 40 ml (20–150 ml) and none of the patients required blood transfusion.

The drainage tube was removed at 2–4 days (median, 3 days) postoperatively, and the median postoperative hospitalization was 7 days (range, 5–9 days). All patients were discharged with a urethral catheter and the catheter was removed at 14 days after surgery. The median indwelling time of the double-J stent was 8 weeks (range, 4–8 weeks). The clinical outcomes are presented in Table 2.

**Table 2:** Clinical outcomes of patients.

Variables	Total
Total number, (n)	27
Length of ureteral defect (cm), median (range)	6 (5–17)
Combined with psoas hitch technique	14 (51.9%)
Operative time (min), median (range)	155 (90–220)
Blood loss (ml), median (range)	40 (20–150)
Postoperative serum creatinine (μmol/L), median (range)	88.00 (42–339)
Postoperative eGFR (ml/min*1.73 m^2^), median (range)	87.04 (13.84–141.15)
Drainage tube removal time (days), median (range)	3 (2–4)
Postoperative hospitalization (days), median (range)	7 (5–9)
Indwell time of double-J stent (days), median (range)	8 (4–8)
Success rate, n (%)	26 (96.3%)
Total complications, Clavien grade classification, n (%)	5 (18.5%)
Fever (> 38 °C) (G I)	2 (7.4%)
Hematuria (G II)	1 (3.7%)
Urinary tract infection required antibiotics (G II)	1 (3.7%)
Stenosis recurrence (G III)	1 (3.7%)
Follow up time (months), median (range)	24 (12–48)

*eGFR* estimated glomerular filtration rate, *G* grade.

The postoperative complications were classified by the Clavien-Dindo grade system^6^. A total of five patients suffered postoperative complications. Two patients suffered from fever (grade I) and were treated with antipyretics. Hematuria (grade II) was observed in one patient who had long-segment ureter defect and was cured with hemostatic. Urinary tract infection required antibiotics (Grade II) was seen in one patient. In one patient with proximal ureteral avulsion, renal colic and nausea occurred after removing the double-J tube at 8 weeks postoperatively, then ureteroscopy was performed, anastomotic edema and short stenosis (Grade III) were seen. Two 7 F double-J stents were inserted and successfully removed 6 months later.

**Figure 2:**
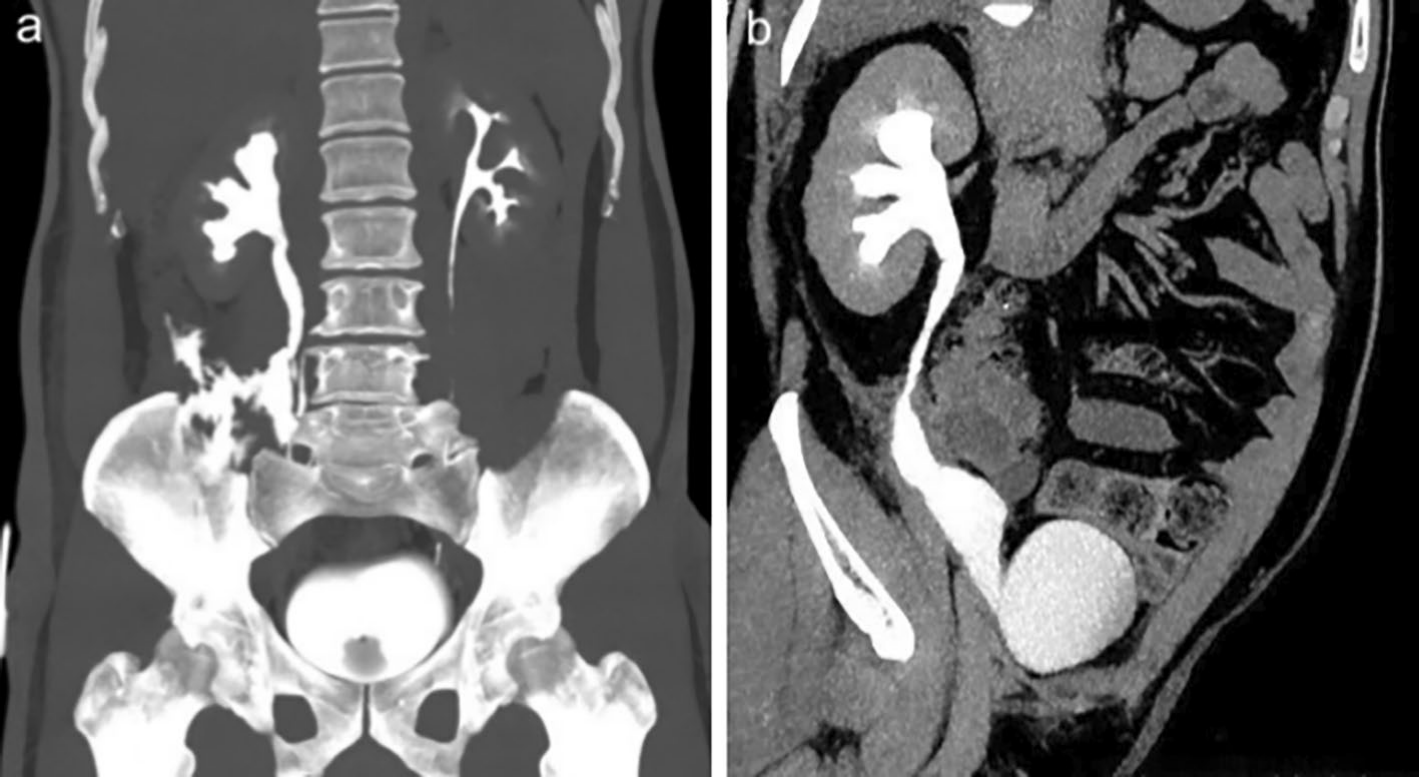
The preoperative and postoperative radiological results. (**a**) preoperative CT urography; (**b**) postoperative CT urography.

All 27 patients underwent physical, laboratory and radiological examinations at 3, 6 and 12 months after surgery (Fig. 2a,b). The median follow-up time was 24 months (range, 12–48 months). Except for the patient described above, no other patient experienced recurrence of ureteral stenosis during this period. Improvement of hydronephrosis were seen in 25 (92.6%) patients, the remaining two patients had hydronephrosis levels comparable to preoperative status, but radiological results indicated good ureteral patency. Preoperative flank or abdominal pain was relieved and other preoperative symptoms disappeared in all patients.

Patients were divided into two groups according to ureteral lesion location, including the distal ureter group (Group A) and the proximal or middle ureter group (Group B). Group B had longer ureteral defect length than that of Group A. The age, gender and operative side of the two groups were comparable and without significant differences. Group B had a longer operative time (177.5 min vs. 145.0 min; P = 0.002) and more blood loss (50 ml vs. 40 ml; P = 0.018) than Group A. No significant difference was observed in postoperative hospitalization and total complication rates between the two groups. The final success rate was 100% in both groups (Table 3).

**Table 3:** Clinical outcomes of distal ureter group and proximal or middle ureter group.

	Distal ureter group	Proximal or middle ureter group	*Z/χ*^*2*^	*P* value
Total number, (n)	15	12		
Age (years), median (range)	46 (26–69)	47 (21–81)	− 0.489	0.639
Gender, n (%)				
Male	4 (26.7%)	6 (50.0%)		0.257*
Female	11 (73.3%)	6 (50.0%)		
Operative side, n (%)				
Left	7 (46.7%)	9 (75.0%)		0.239*
Right	8 (53.3%)	3 (25.0%)		
Length of ureteral defect (cm), median (range)	6 (5–6)	13 (7–17)	− 4.504	0.000
Operative time (min), median (range)	145.0 (90–180)	177.5 (120–220)	− 3.110	0.002
Blood loss (ml), median (range)	40 (20–90)	50 (30–150)	− 2.342	0.018
Postoperative hospitalization (days), median (range)	7 (5–8)	7 (5–9)	− 0.962	0.336
Total complications, n (%)	1 (6.7%)	4 (33.3%)		0.139*
Success rate, n (%)	15 (100.0%)	11 (91.7%)		0.444

*Fisher’s exact test.

## Discussion

The ureter is well protected in the retroperitoneal space and is not susceptible to damage. Iatrogenic factor is the most common etiology of ureteral injury, and gynecological surgeries account for the largest proportion^7^, followed by urological procedures^8^. However, with the increasing popularity of endoscopic procedure and laparoscopic surgery, the proportional distribution of iatrogenic factors has changed^9^. In our study, urological surgery was the most common cause, followed by gynecological surgery. The majority of urological etiology was ureteral holmium laser lithotripsy, and laparoscopic hysterectomy was the most common gynecological etiology. Our result was similar to the previous study^9^.

The time to diagnosis of ureteral injury is an important factor for therapeutic options. However, 50–70% of ureteral injuries failed to be clarified intraoperatively in time^10^. Extensive ureteral perforation or avulsion can be detected during endoscopic or laparoscopic surgery, and immediate surgical repair is available^2^, however, minor perforation, mucosal abrasion and non-transversal injury are often ignored or undiscovered. Symptoms resulting from ureteral injuries include flank or abdominal pain, fever, hematuria, and urinary leakage^11^. The occurrence of these manifestations indicates the possibility of ureteral injury. Delayed diagnosis of ureteral injury may lead to ureteral stenosis or atresia, ureteric dilatation, hydronephrosis, urinary tract infection, and even progressive impairment of renal function^12^, which may need further intervention.

Currently, the ideal time for reconstructive surgery remains controversial^13^. Ambani et al. compared early (≤ 7 days) repair with delayed (> 7 days) repair of the injured ureter, they found no difference in outcomes between the two groups^13^. El-Abd et al. *s*ummarized their experience with immediate and late management of ureteral injuries, they found better long-term outcomes in the immediate management group^14^. A retrospective study of 12 patients with ureteric injuries during gynecologic surgery conducted by Han et al. showed that early recognition and management could prevent further morbidity^15^. Lee et al. demonstrated that ureteral rest, defined as no hardware crossing the ureter stenosis for ≥ 4 weeks, was associated with a higher reconstruction success rate and less blood loss^16^. In our study, patients underwent reconstructive surgery once the diagnosis was clear, rather than upon further waiting. For patients who were inappropriate for immediate surgery, a percutaneous nephrostomy tube was highly recommended. In addition to draining renal urine and protecting renal function, the more important role of the nephrostomy tube was to maintain ureteral rest and facilitate the identification of the stenotic segment during surgery.

In addition to the etiology and time of diagnosis, treatment protocols are related to other factors such as the characteristics of injury, the location of injured ureter, the patient status, and surgeon experience^17^. Compared to the proximal and middle ureter, the distal ureter is more susceptible to damage^1,15,17^. The distal ureter was also the most common injury site in our study. Endoscopic surgery is suggested for patients with short ureteral stenosis (< 2 cm)^3^. For long segment of ureteral stenosis, formal ureteral reconstructive procedures remain the gold standard treatment^3,18^. Ureteroneocystostomy is suitable for distal ureteral defect up to 4–5 cm in length. Psoas bladder hitch surgery could repair 5–8 cm gap above the ureteric orifice^19^. Boari bladder flap is usually used for bridging long defect up to 15cm^20,21^. Bai et al.reported their laparoscopic reconstructive experience with bladder muscle flap for treating full-length ureteral defect, and the longest length was 21 cm^22^. In our study, the median defect length was 6 cm, ranging from 5 to 17 cm. The bladder flap technique has been successfully utilized to bridge long-length ureteral defect above the ureteral orifice. However, for relatively longer ureteral defect, we combined bladder flap with PH technique to reduce anastomotic tension and stabilize the bladder.

Compared with open surgery, laparoscopic reconstructive technique superior in terms of less blood loss, fewer complications, and better cosmetic outcomes^1,23^. Numerous studies have demonstrated satisfactory outcomes and acceptable complications of laparoscopic bladder flap surgery ^21,22,24,25^. Laparoscopy has specificities of magnifying effect and extremely clear vision, which could avoid excessive dissection of the healthy ureter, achieve effect of mucosa -mucosa anastomosis and maximize protection of the ureteral blood supply. However, long ureteric defect still presents a challenge for urologists, so senior surgeons with rich experience in reconstructive surgery and laparoscopic skills are recommended. In our study, all reconstructive surgeries were successfully completed via laparoscopic approaches, and none were converted to open surgery or underwent other reconstructive surgeries. We compared the clinical outcomes of patients with different ureteral defect lengths. Long ureteral defect was associated with longer operative time and more blood loss, which was related to harvesting longer flap and consuming more time during the ureteroplasty process. However, the postoperative hospitalization, total complications rate and total success rate between short and long defect groups had no significant difference. Therefore, LUCBF was effective for long segmental defects, but it was best for experienced surgeons to perform this procedure.

The principles of laparoscopic reconstructive surgery are consistent with those of open approaches and include tension-free, watertight and mucosa–mucosa anastomosis, gentle manipulation of the ureter, maximal protection of ureteric vascularization, and reinforcement of the blood supply^1,12^. Based on these principles, several technical difficulties must be considered. First, the hard and cicatricial tissue surrounding the ureter, which we call "ureteral armor", must be completely excised until the pink healthy ureter proximal to the stenotic segment appeared, which was a key step in minimizing the possibility of recurrent stenosis. Residual scar tissue may interfere with incision healing and eventually lead to anastomotic leakage or poor healing. On the other hand, excessive ureteral dissection must be avoided and care should be taken to protect the intrinsic ureteral blood supply^4^. Then, the healthy ureter was freed for at least 2 cm long and cut longitudinally at the posterior wall, which was prepared for anastomosis. Second, several studies have reported the application of oblique^22^, spiraled^26^, trapezoidal or S-shaped^1^ bladder flaps in the reconstruction of ureteric defects. The different shapes were designed based on preoperative bladder capacity and morphology, with the aim of achieving tension-free anastomosis and maintaining adequate blood supply. Since the blood supply decreased from the base to the apex of the flap, we harvested flap with sufficiently broad base that was long enough for tension-free anastomosis. The length of the flap must be 2 cm longer than the ureteral defect, so that it could achieve the vertex of the incised ureteral posterior wall can be reached for tension-free anastomosis. The width of the bladder flap was 4–5 cm at the base and 2–3 cm at the tip, and the length/width ratio was 3–4:1, which was similar to the principles described in previous studies^1,21^. Third, the midpoint of the flap apex was anastomosed to the vertex of the incised ureteral posterior wall, and interrupted side-to-side anastomosis was used for both the edges of the bladder flap and the ureter. A trumpet-shaped anastomosis was formed to ensure the intraluminal patency. Finally, the ureter-flap anastomosis, tubularized flap and bladder incision were enclosed by pedicled omentum for extra blood supply. The omental wrapping had the functions of revascularization, tissue regeneration, promotion of wound healing and anti-infection^27^, which was especially suitable for reconstructive surgery.

The recurrence of stenosis is a serious postoperative complication, and most cases occur within the first year after surgery^28^; therefore, it is suggested that all patients be followed for at least 12 months. Currently, no standard protocols for postoperative surveillance exist. Ghosh et al. formulated a strict radiographic surveillance system, including the application of IVU at 3 months postoperatively, and ultrasonography was performed 6-monthly for 2 years^28^. However, we paid more attention to the follow-up protocols, and postoperative imaging was utilized at every 3 months within the first year. During the follow-up period, only one patient suffered stenosis recurrence and this patient was cured with prolonged double-J stent placement. None of the remaining patients in our study had recurrent stenosis or increased hydronephrosis.

The indications for bladder flap in our study included ureteral stenosis or avulsion, ureterogenital fistula, and ureteral polyp. In addition to benign ureter lesions, bladder flap could also be performed for treating transitional cell carcinoma^29^. With the popularity of minimally invasive techniques, LUCBF has been performed by several centers for various indications, as depicted in Table 4^4,21,22,25,30^. Our outcomes were equivalent to the results of these studies.

**Table 4:** Reports of laparoscopic ureteral ureteroneocystostomy with bladder flap.

Study	Cases	Stenosis aetiology	Ureter defect (cm)	Gender M/F	Side L/R	MOT (min)	MBL (ml)	MPH (days)	MFU (month)	Complications	Success rate
Our study	27	15, urological surgery 8, gynecological surgery 3, ureteral polyp 1, impacted calculi	6 (5–17)	10/17	16/11	155 (90–220)	40 (20–150)	7 (5–9)	24 (12–48)	2, fever 1, hematuria 1, urinary tract infection 1, Stenosis recurrence	96.3%
Zhang G^21^ (2022)	8	3, urologic surgery 1, gynecological surgery 1, appendicitis operations 1, uterine radiotherapy 1, ureteral neoplasms 1, endometriosis	7.94 (4–15)	2/6	4/4	180 (120–240)	32.5 (20–50)	6 (4–7)	6–12	1, occasional abdominal pain	100%
Wu Y^4^ (2021)	6	4, congenital causes 1, iatrogenic injury 1, inflammation	2.0 (1.5–3.0)	1/5	1/5	193.3 (160–270)	41.5 (10–58)	8.2 (6–11)	24.5 (14–29)	no	83.3%
Bai Y^22^ (2021)	10	10, iatrogenic ureteral injuries	18.9 (14–21)	3/7	4/6	124 (89–220)	92.2 (45–158)	10.5 (7–14)	18.5 (3–39)	1, postoperative anastomotic stricture	90%
Singh M^25^ (2018)	7	5, urolithiasis 2, ureteral tumor	NA	5/2	1/6	182 (115–250)	175 (120–250)	3 (2–7)	19.7 (6–45)	1, External iliac vein injury	100%
Castillo^30^ (2013)	30	16, gynecological surgery 14, urological surgery	7 (5–20)	8/22	13/17	161 (90–280)	123 (0–500)	4.8 (2–10)	32 (5–60)	1, adynamic ileus 1, urinary leakage 1, uroperitoneum 1, hemoperitoneum 1, bladder flap stenosis	96.6%

*M/F* male/female, *L/R* left/right, *MOT* mean/median operative time, *MBL* mean/median blood loss, *MPH* mean/median postoperative hospitalization, *MFU* mean/median follow-up.

Our study had several limitations. The main limitation was that this was a retrospective study in a single center, selective bias could not be avoided. Second, a control group was lacking and the total sample was small. For further study, a multicenter prospective study with larger sample size is recommended.

## Conclusion

LUCBF is a safe and feasible technique for treating benign ureteral stenosis. Long ureter defect was related to longer operative time and more blood loss.

### Supplementary Information

Supplementary Information.

## Data Availability

All data generated or analysed during this study are included in this published article (and its Supplementary Information files).
